# Assessing the Porosity of Denture Base Acrylic Resin Loaded With Fluconazole

**DOI:** 10.1155/ijod/7257953

**Published:** 2025-10-31

**Authors:** Heba Alajami, Alaa'a Salloum

**Affiliations:** Department of Removable Prosthodontics, Faculty of Dentistry, Damascus University, Al-Mazzeh Street, P.O. Box 30621, Damascus, Syria

**Keywords:** acrylic resin, denture, fluconazole, porosity, surface roughness

## Abstract

**Objectives:**

The study aimed to evaluate the effect of adding fluconazole pure (FLUp) 10% and fluconazole from capsules (FLUcap) 25% on the porosity of the heat-cured acrylic resin.

**Methods:**

This study included 30 disc-shaped acrylic samples with a diameter of 30 mm and a thickness of 3 mm. Three subgroups: 10 pieces representing the control group without adding fluconazole, 10 pieces representing acrylic resin modified with FLUp 10%, 10 pieces representing acrylic resin modified with FLUcap 25%. The porosity was analyzed using the gravimetric method based on Archimedes' principle, by weighing the sample in the air and in the water to calculate both the dry and wet sample volumes. The measurement was done in two-time stages: T1 before immersion of the samples in distilled water, and T2 after immersion in distilled water for 28 days at a temperature of (37 ± 2)°C.

**Results:**

The Mann–Whitney test revealed no significant differences in porosity averages among the three studied groups in both time stages T1 and T2 (*p* > 0.05). The Wilcoxon test showed that porosity in the FLUp group remained statistically unchanged between T1 and T2 (*p* > 0.05). In contrast, the FLUcap group exhibited a significant differences in porosity between T1 and T2 (*p* < 0.05).

**Conclusion:**

The addition of both FLUp and FLUcap did not affect the porosity of the heat-cured acrylic resin.

**Clinical Significance:**

This study will contribute to the field of antimicrobial dental materials, whereas fluconazole-modified acrylic resin may be considered a promising treatment for denture-induced stomatitis and this study is considered the foundation of future clinical research.

## 1. Introduction

Denture-induced stomatitis presents a prevalent clinical challenge, with a reported incidence ranging from 20% to over 80% among elderly denture wearers [1]. This condition is frequently associated with the prolonged use of ill-fitting dental prostheses. Standard management protocols combine antifungal therapy, administered either topically (e.g., nystatin, clotrimazole, and miconazole) or systemically (e.g., fluconazole and ketoconazole), with diligent denture maintenance, including disinfection, nocturnal removal, and procedures such as modification, relining, or reconstruction [2, 3]. A significant limitation of topical treatments is the rapid clearance of the active agent from the oral cavity by salivary flow [4], and critically, systemic antifungals demonstrate limited efficacy without concurrent topical application [5].

Treatment complexity escalates in elderly patients and those with debilitating conditions or impaired motor skills, who often struggle to maintain adequate oral hygiene and adhere to strict medication regimens [6]. While alternative disinfection methods offer a strategy to avoid systemic antifungals and their associated risks, including gastrointestinal disturbances, hypersensitivity, hepatonephrotoxicity, and drug interactions [3]. Although disinfection methods considered as an alternative to antifungal drugs in treating stomatitis, it also has minor side effects such as pigmentation and an altered sense of taste [7, 8].

Several researchers studied the possibility of improving the antibacterial effect of denture bases by incorporating antimicrobial agents into the acrylic resin matrix [9]. This approach ensures sustained and localized drug delivery directly to the infection site, independent of patient compliance, while mitigating systemic side effects and drug interactions [10]. Numerous studies have explored this concept by embedding antifungals into denture bases, soft liners, and tissue conditioners, enabling a controlled release that inhibits microbial colonization [11, 12].

Fluconazole, a broad-spectrum antifungal agent developed in the 1980 s and widely used for systemic and prophylactic care in patients at high risk of fungal infection (cancer chemotherapy, immunosuppressed transplant patients) [13], is a prime candidate for such modifications. The success of any denture base modification depends on its impact on the material's physical properties. A critical property is surface roughness and porosity, which are often consequences of suboptimal fabrication techniques as rough wax pattern, coarse powder particles of the investing material, high water/powder ratio and inadequate mixing of plaster stone, air trapping due to inadequate vibration of the plaster mix, incomplete dewaxing, improper application of separating medium, late or too early packing—before or after the dough stage is reached, and lack of pressure during packing or curing [14].

Porosity, the formation of internal and surface voids, severely compromises a prosthesis's physical, esthetic, and hygienic properties. Each pore is considered an area of stress concentration that reduce fracture resistance and serves as a reservoir for the microorganisms, which promotes biofilm adhesion leading to mucosal irritation. Porosity is likely to develop in thicker portions of a denture base, due to the vaporization of unreacted monomers when the temperature of the resin reaches the boiling point [15].

Therefore, this in vitro study aims to evaluate the effect of incorporating 10% pure fluconazole powder and 25% fluconazole capsule content (equivalent doses based on bioassay) on the porosity of heat-cured polymethyl methacrylate (PMMA) [12]. The null hypothesis was: there is no significant difference in the porosity of heat-cured PMMA with, and without fluconazole pure (FLUp) (10%) and fluconazole capsules (25%), respectively.

## 2. Materials and Methods

### 2.1. Sample Preparation

The sample size was calculated using G^*⁣*^*∗*^^Power 3.1.9.4 depending on a previous study [16].

The effect of the incorporation of FLUp or fluconazole from capsules (FLUcap) on the porosity of PMMA was tested in vitro.

For the analysis of porosity, 30 disc-shaped acrylic samples with a diameter of 30 mm and thickness of 3 mm (*n* = 10) were made, where a rubber frame with the same dimensions as the samples. Whereas, a rubber ring in the same dimension as the specimens were used to deflask the samples easily and to dispense the need for wax models and the need to break plaster.

**Figure 1:**
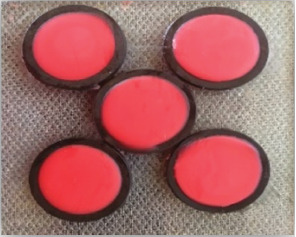
Wax poured into the frames.

**Figure 2:**
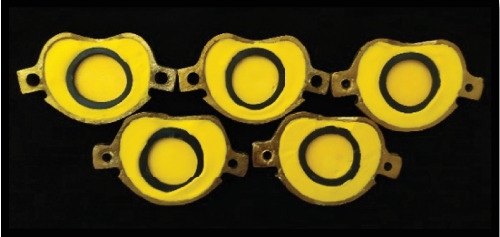
Plaster molds.

Wax was poured into the frames (Figure 1), then they were invested with plaster, and the flask was placed in a water bath at a temperature of 100°C for 20 min, then the flask was opened and the two halves of it were placed in the water bath again for 5 min to ensure no wax residue were left, thus obtaining molds within plaster that can be repacked with acrylic dough several times (Figure 2).

Then the discs were allocated into two different groups as follows:

(T1) discs were tested immediately

(T2) after discs were stored in distilled water for 28 days at 37°C.

Wax was poured into the rings and invested with plaster inside the flask, then it was placed in a water bath at a temperature of 100°C for 20 min, then the flask was opened and the two halves of it were placed in the water bath again for 5 min to ensure that no wax was left.

**Figure 3:**
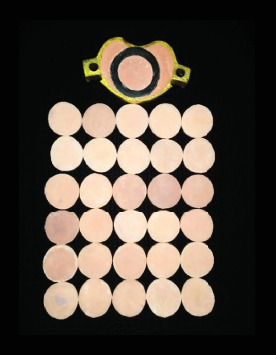
Acrylic specimens after deflasking.

Acrylic excess was removed from the final acrylic specimens using a tungsten carbide bur and finished using (grit-400) waterproof abrasive paper (Figure 3).

### 2.2. Testing

The porosity test was done in two-time stages:

T1: Immediately after curing the sample, before immersing them in distilled water.

T2: After immersing the samples for 28 days at a temperature of (37 ± 2)°C.

To minimize the measuring bias, all the groups were tested by two blinded assesors under the same environmental conditions.

Porosity was related to the amount of absorbed water by each specimen after storing it in distilled water at 37°C. The samples were weighed on a digital analytical balance (three decimal places Sartorius GMBH Gottingen, German) in two stages. In the first one, the specimen was daily weighed after a storage period in a desiccator (Vidrolabor, São Paulo, SP, Brazil). In the second one, the specimen was also daily weighed after a storing period in distilled water in an oven (Memmert U15 oven, Western Germany) at 37°C. The final record of weighing for dry and wet specimens was weighed as they reached a stable mass, evidenced after stabilization in a milligram scale.

The porosity was analyzed using the gravimetric method based on Archimedes' principle. An additional weighing was performed after each stage with the specimen immersed in distilled water. The porosity was calculated according to the following equations:

(1) $$\begin{array}{c} \text{Vs dry}=\left(\text{md–}{\text{ md}}^{\prime }\right)/\rho \text{water}, \end{array}$$

(2) $$\begin{array}{c} \text{Vs wet}=\left(\text{mw–}{\text{ mw}}^{\prime }\right)/\rho \text{water}, \end{array}$$

(3) $$\begin{array}{c} \text{Porosity}\%=100\times \left(\text{Vs wet}-\text{Vs dry}\right)/\text{ Vs dry}, \end{array}$$

where Vs dry (mL) is the volume of the dry specimen, md (g) is the mass of the dry specimen recorded in the air, md' (g) is the mass of the dry specimen recorded with the specimen immediately immersed in water, *ρ*water (g/mL) is the density of water, Vs wet (mL) is the volume of the wet specimen, mw (g) is the mass of the wet specimen recorded in the air, and mw' (g) is the mass of the wet specimen recorded with the specimen immediately immersed in water.

## 3. Results

**Figure 4:**
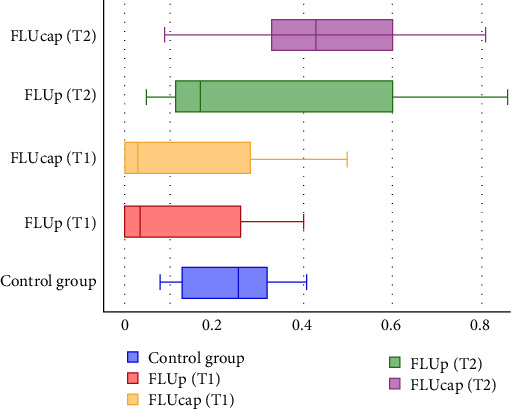
Boxplot illustrating the distribution of porosity values, showing the median and interquartile range (IQR) for each group.

The median and interquartile range (IQR) for the porosity measurements of all experimental groups are presented in (Table 1) and visually summarized in (Figure 4).

The normality of distribution of porosity data was assessed using the Kolmogorov–Smirnov test (Table 2). As the assumption of normality showed that the porosity data in two out of five groups deviated from a normal distribution. Thus, parametric tests were not performed.

Intergroup comparisons at the initial time point (T1), conducted using the Mann–Whitney *U* test, revealed a statistically significant difference in porosity between the Control and FLUcap groups (*p* ≤ 0.001). No significant difference was observed between the Control and FLUp groups (*p* = 0.067) or between the FLUcap and FLUp groups at T1 (*p* = 0.11). This pattern of significance remained consistent at the T2 time point, with a significant difference between the Control and FLUcap groups (*p* = 0.011) and no significant difference found between the other group pairings (Control vs FLUp: *p* = 0.880; FLUp vs FLUcap: *p* = 0.089) (Table 3).

Intragroup analysis of porosity changes over time, was performed using the Wilcoxon test, indicated that immersion in water had a divergent effect depending on the formulation. No statistically significant difference was found between the pre- (T1) and postimmersion (T2) porosity values for the FLUp group (*p* = 0.139). In contrast, a statistically significant increase in porosity was observed for the FLUcap group after immersion (*p* = 0.005) (Table 4).

## 4. Discussion

The pursuit of enhancing the biological properties of denture base acrylic resin (DBR) has led to the investigation of various antimicrobial additives. This strategy offers the significant advantage of providing localized drug delivery directly at the infection site, thereby operating independently of patient compliance and minimizing risks of systemic side effects and drug interactions [9, 10]. While the development of antimicrobial DBR has been extensively explored, a comprehensive understanding of how these incorporated agents, particularly antifungals, impact the material's critical physical properties remains comparatively limited.

Fluconazole has emerged as a promising candidate for such modifications, demonstrating superior inhibitory effects against *Candida albicans* compared to nystatin and miconazole [17], and exhibiting effective release profiles from acrylic resins even after heat-curing processes [18, 19]. Consequently, this study focused on evaluating the effect of fluconazole incorporation on the porosity of the acrylic resin, which profoundly influences the mechanical integrity, esthetic quality, and hygiene of dentures, as surface and internal pores act as a reservoir for plaque biofilm, a primary factor in the recurrence of denture stomatitis [15], and adding fluconazole will help to close these pores initially and then it will leach into the affected mucosa under the denture [19]. However, it might leave pores and increase porosity after being leached and that is what was studied in this research, where, 10% of the PMMA powder was replaced with pure fluconazole powder in FLUp group and 25% was replaced with a pharmaceutical capsule formulation in FLUcap group. The higher concentration in the FLUcap group was designed to deliver a bioequivalent antifungal dose, as determined by bioassay tests indicating the minimum inhibitory concentration (MIC) for the pure form was 2.5 times that of the drug form [12].

The initial findings revealed that both modified groups exhibited lower porosity than the control group at baseline (T1). This suggests that the incorporated powders, irrespective of form, primarily functioned as fillers within the PMMA matrix. The particles likely occupied spaces within the polymer network that would otherwise evolve into voids during polymerization. The significantly lower porosity in the FLUcap group is logically attributed to its higher filler content, which includes not only the active drug but also excipients.

However, immersion in water for 28 days (T2) provided critical insight into the stability of these composites. The lack of a statistically significant change in porosity for the FLUp group indicates that the pure fluconazole particles are well-integrated and stable within the resin, resisting dissolution, and thus preserving the structural integrity of the matrix over time. In contrast, the FLUcap group exhibited a statistically significant increase in porosity postimmersion. We postulate that this is a direct result of the leaching of water-soluble excipients from the pharmaceutical formulation. This process creates new microvoids, effectively transforming the additive's role from a static filler to a dynamic, leachable component that can compromise the long-term structural architecture.

These results found support in the existing literature. The initial filler effect aligns with studies incorporating solid additives like silver zeolite [20] or zirconium oxide nanofillers [21], which reduced the porosity and increased the density of the acrylic resin. However, the composition of the additive is paramount. Unlike solid fillers, a composite drug formulation containing soluble excipients can undermine the resin's structural stability through leaching, a phenomenon not typically associated with additives like silver or zirconium oxide. The stability of the studied pure fluconazole group is consistent with findings for other antimicrobials in soft liners [22], reinforcing that a well-integrated pure drug can be retained effectively by the polymer matrix. Most importantly, despite the observed changes, the final porosity values for all groups remained substantially below the 11% threshold considered clinically acceptable [23, 24]. This indicates that both fluconazole formulations can be utilized without affecting this vital physical property.

A primary limitation of this study is the use of Archimedes' principle for porosity measurement [25]. While effective for quantifying total void volume, this method cannot characterize the morphology, size, and distribution of the pores.

Future research should employ microscopic techniques (e.g., scanning electron microscopy) to characterize this pore architecture in detail. Furthermore, as an in vitro study, there is a need for subsequent clinical trials to validate the long-term stability, antifungal efficacy, and biocompatibility of these modified resins in the complex oral environment.

## 5. Conclusion

This study demonstrates that the modification of heat-cured acrylic resin with fluconazole at concentrations of 10% (pure form) and 25% (drug form), yielded porosity levels thats were not significantly compromised and were within the established range for clinical acceptability, supporting their potential utility in fabricating antimicrobial dental prostheses.

## Figures and Tables

**Table 1:** Descriptive statistics of porosity (%) across the tested groups.

Period	Group	Median	IQR
	Control group	0.255	0.19

T1	Fluconazole pure (FLUp) group	0.035	0.26
	Fluconazole from capsules (FLUcap) group	0.03	0.28

T2	Fluconazole pure (FLUp) group	0.15	0.46
	Fluconazole from capsules (FLUcap) group	0.43	0.27

**Table 2:** Results of the Kolmogorov–Smirnov normality test.

Period	Group	*p*-Value	Significance
	Control group	0.200	Normal

T1	Fluconazole pure (FLUp) group	**0.018**	Paranormal
	Fluconazole from capsules (FLUcap) group	**≤0.001**	Paranormal

T2	Fluconazole pure (FLUp) group	0.200	Normal
	Fluconazole from capsules (FLUcap) group	0.200	Normal

*Note:* Bold *p*-values indicate statistical significance (*p* < 0.05).

**Table 3:** Results of the Mann–Whitney test for intergroup comparisons of porosity at T1 and T2.

*p*-Value	*Z*-value	Mann–Whitney value	Studied groups		Period
**0.067**	**−1.83**	**26**	FLUp	Control group	T1
**≤0.001**	**−3.556**	**4.5**	FLUcap		
0.11	**−1.597**	**32**	FLUcap	FLUp	

0.88	**−** **0.151**	**48**	FLUp	Control group	T2
**0.011**	**−2.534**	**16.5**	FLUcap		
0.089	**−1.701**	**27.5**	FLUcap	FLUp	

*Note:* Bold *p*-values indicate statistical significance (*p* < 0.05).

**Table 4:** Results of the Wilcoxon test for intragroup comparisons of porosity before (T1) and after (T2) immersion.

*p*-Value	*Z*-value	Studied groups
0.139	**−1.481**	FLUp (T1)
		FLUp (T2)

**0.005**	**−2.805**	FLUcap (T1)
		FLUcap (T2)

*Note:* Bold *p*-values indicate statistical significance (*p* < 0.05).

## Data Availability

The datasets generated and analyzed during the current study are available from the corresponding author upon request.

## References

[1] Gendreau L., Loewy Z. G. (2011). Epidemiology and Etiology of Denture Stomatitis. *Journal of Prosthodontics-Implant Esthetic and Reconstructive Dentistry*.

[2] McReynolds D. E., Moorthy A., Moneley J. O., Jabra-Rizk M. A., Sultan A. S. (2023). Denture Stomatitis—An Interdisciplinary Clinical Review. *Journal of Prosthodontics-Implant Esthetic and Reconstructive Dentistry*.

[3] Mylonas P., Milward P., McAndrew R. (2022). Denture Cleanliness and Hygiene: An Overview. *British Dental Journal*.

[4] Martins K. V., De Lacerda Gontijo S. M. (2017). Treatment of Denture Stomatitis: Literature Review. *Brazilian Journal of Dentistry*.

[5] Cawson R. A., Odell E. W. (2017). *Cawson’s Essentials of Oral Pathology and Oral Medicine*.

[6] Nadendla L. K., Reddy M. R. (2013). Denture Stomatitis—A Review. *Indian Journal of Dental Advancements*.

[7] Emami E., Kabawat M., Rompre P. H., Feine J. S. (2014). Linking Evidence to Treatment for Denture Stomatitis: A Meta-Analysis of Randomized Controlled Trials. *Journal of Dentistry*.

[8] Herrera D. (2013). Chlorhexidine Mouthwash Reduces Plaque and Gingivitis. *Evidence-Based Dentistry*.

[9] An S., Evans J. L., Hamlet S., Love R. M. (2021). Incorporation of Antimicrobial Agents in Denture Base Resin: A Systematic Review. *The Journal of Prosthetic Dentistry*.

[10] Douglas W. H. (1977). Dental Materials as Carriers for Therapy. *Dental Update*.

[11] Bettencourt A. F., Costa J., Ribeiro I. A. (2023). Development of a Chlorhexidine Delivery System Based on Dental Reline Acrylic Resins. *International Journal of Pharmaceutics*.

[12] Salim N., Moore C., Silikas N., Satterthwaite J. D., Rautemaa R. (2012). Fungicidal Amounts of Antifungals are Released From Impregnated Denture Lining Material for up to 28 Days. *Journal of Dentistry*.

[13] Herrera S., Acosta J. (2013). Fluconazole Pharmacology, Clinical Uses and Health Effects. *Nova Science Publishers*.

[14] Shama Bhat V., Nandish B. (2015). *Science of Dental Materials Clinical Applications*.

[15] Anusavice K. J., Shen C., Rawls H. R. (2013). *Phillips’ Science of Dental Materials*.

[16] Figuerôa R. M. S., Conterno B., Arrais C. A. G., Sugio C. Y. C., Urban V. M., Neppelenbroek K. H. (2018). Porosity, Water Sorption and Solubility of Denture Base Acrylic Resins Polymerized Conventionally or in Microwave. *Journal of Applied Oral Science*.

[17] Pharande A., Chopde N., Khade M. N., Khadtare Y. R., Shah S. S., Apratim A. (2012). In Vitro Antifungal Activity of Two Tissue Conditioners Combined With Nystatin, Miconazole and Fluconazole Against *Candida albicans*. *The Journal of Contemporary Dental Practice*.

[18] Chincholikar S., Sabane A. V., Patel A. (2019). Comparative Evaluation of Two Antifungal Agents Incorporated in Auto Polymerising Denture Base Resin, Heat Polymerising Denture Base Resin and Permanent Silicone Soft Liner-An In Vitro Study. *Journal of Clinical and Diagnostic Research*.

[19] Amin W. M., Al-Ali M. H., Salim N. A., Al-Tarawneh S. K. (2019). A New Form of Intraoral Delivery of Antifungal Drugs for the Treatment of Denture-Induced Oral Candidosis. *European Journal of Dentistry*.

[20] Kartika U. K., Agrawal B., Yadav N. S., Singh P. P., Rahangdale T. (2015). The Effect of Microwave Processing and Use of Antimicrobial Agent on Porosity of Conventional Heat Cured Denture Base Resin: An In Vitro Study. *The Journal of Indian Prosthodontic Society*.

[21] Mohammed D. H., Mudhaffar M. (2012). Effect of Modified Zirconium Oxide Nano-Fillers Addition on Some Properties of Heat Cure Acrylic Denture Base Material. *Journal of Baghdad College of Dentistry*.

[22] Lima J. F. M., Maciel J. G., Arrais C. A. G., Porto V. C. (2016). Porosity of Temporary Denture Soft Liners Containing Antifungal Agents. *Journal of Applied Oral Science*.

[23] Council on Dental Materials and Devices (1975). Revised American Dental Association Specification No. 12 for Denture Base Polymers. *The Journal of the American Dental Association*.

[24] Jerolimov V., Brooks S. C., Huggett R., Bates J. F. (1989). Rapid Curing of Acrylic Denture-Base Materials. *Dental Materials*.

[25] Yannikakis S., Zissis A., Polyzois G., Andreopoulos A. (2002). Evaluation of Porosity in Microwave-Processed Acrylic Resin Using a Photographic Method. *The Journal of Prosthetic Dentistry*.

